# Ongoing outbreak with well over 4,000 measles cases in Italy from January to end August 2017 − what is making elimination so difficult?

**DOI:** 10.2807/1560-7917.ES.2017.22.37.30614

**Published:** 2017-09-14

**Authors:** Antonietta Filia, Antonino Bella, Martina Del Manso, Melissa Baggieri, Fabio Magurano, Maria Cristina Rota

**Affiliations:** 1 Department of Infectious Diseases, Istituto Superiore di Sanità, Rome, Italy; 2 These authors contributed equally to this article and share first authorship; 3 National Reference Laboratory for Measles and Rubella, Istituto Superiore di Sanità, Rome, Italy

**Keywords:** Italy, air-borne infections, viral infections, measles, measles-mumps-rubella (MMR) vaccine, surveillance, epidemiology

## Abstract

We report an ongoing measles outbreak in Italy, with over 4,400 cases reported in 20 Regions from January to August 2017. Median age was 27 years, 88% of the cases were unvaccinated. The highest incidence was in infants below one year of age and 7% of cases occurred among healthcare workers. Three deaths occurred and two cases of encephalitis were reported. Wide immunity gaps and nosocomial transmission are major challenges to measles elimination in Italy.

Measles is targeted for elimination in Italy as in the World Health Organization (WHO) European Region, however, Italy remains one of the 14 countries in the Region with ongoing endemic transmission [1]. In January 2017, the Italian National Health Institute (Istituto Superiore di Sanità-ISS) detected an increase in the number of measles cases reported to the national measles and rubella integrated surveillance system, compared with previous months and years.

We describe characteristics of cases reported in the first 8 months of 2017, main transmission settings, and control measures implemented.

## Outbreak description

**Figure 1:**
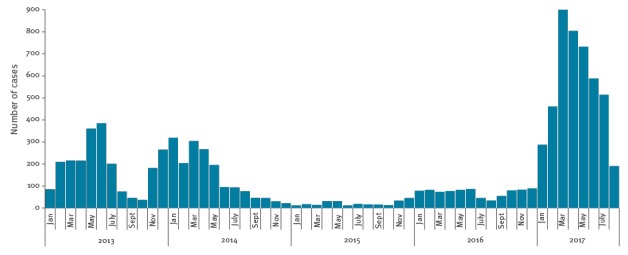
Reported measles cases by month of rash onset, Italy, January 2013−August 2017 (n=9,559)

Between 1 January and 31 August 2017, 4,477 cases were reported to the surveillance system, of which 3,417 (76.3%) were laboratory confirmed (3,189 in WHO-accredited laboratories), 456 (10.2%) were probable and 604 (13.5%) were possible cases according to the European Union (EU) case definition [2]. Figure 1 shows the distribution of measles cases by month of rash onset from January 2013. The peak number of reported cases was reached in March 2017, with 900 cases reported. The outbreak is ongoing at the time of writing this article.

**Figure 2:**
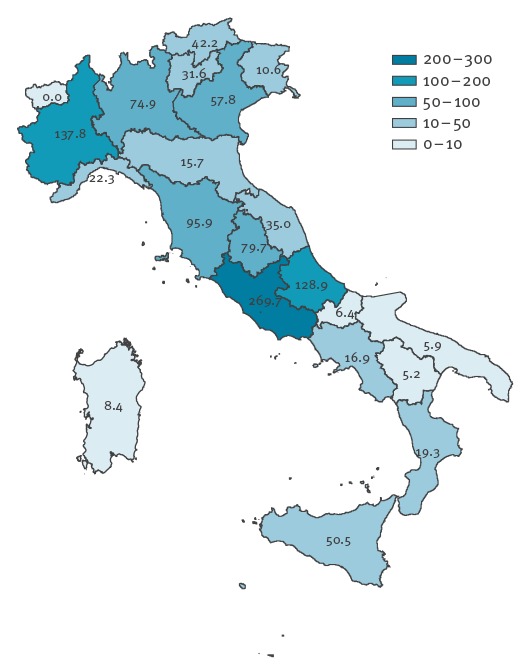
Incidence per 1,000,000 population of reported measles cases by Region, Italy, January−August 2017

The current outbreak affected 20 of the 21 Italian administrative regions but 4,015 (90%) of the cases were reported by only seven regions. The Lazio region, in central Italy, reported the highest number of cases (n = 1,588) and the highest incidence (269.7/1,000,000 population) (Figure 2).

Median age was 27 years (range 25 days–84 years) and 2,270 (50.7%) of the cases were female. Most cases (n = 3,301; 73.8%) were above 15 years of age, but the highest incidence occurred in children aged under one year (Table 1).

**Table 1:** Number and incidence per 1,000,000 age-specific population of reported measles cases, by age group, Italy, January−August 2017

Age group (years)	Number	Percentage	Incidence (per 1,000,000 population)
< 1	253	5.7	541.0
1–4	543	12.1	267.0
5–14	378	8.4	66.5
15–39	2,525	56.4	152.7
> 39	776	17.3	21.6
**Total**	**4,475^a^**	**100.0**	**73.9**

^a^ Information on age was not available for two cases.

Vaccination status was known for 93.4% of cases (n = 4,182), of whom 88.3% (n = 3,691) were unvaccinated, 6.5% (n = 271) had received only one dose of measles-containing vaccine, 1.6% (n = 69) were fully vaccinated and 3.6% (n = 151) had received an unknown number of doses.

The probable transmission setting is known for 30.2% (n=1,352) of the cases. Transmission occurred in families (64.1%; n = 867), nosocomial settings (22.3%; n = 301), schools (10.1%; n = 137) and nomadic settlements (3.5%; n = 47).

Two hundred ninety-six cases (6.6%) occurred among healthcare workers (HCWs), defined as any hospital or other healthcare staff having regular contact with patients. Cases among HCWs were reported from 16 Regions. Median age was 33 years (range: 19–57 years). Vaccination status of HCWs was reported in 94.9% of cases (n = 281): 239 (85.1%) were not vaccinated, 28 (10.0%) had received one dose, 8 (2.8%) two doses, 6 (2.1%) could not recall the number of doses received.

Genotypes B3, D8 and H1 were identified: B3 and D8 represent endemic genotypes, while genotype H1 was identified only in one small outbreak at the beginning of the year.

Complications were reported by 35.1% (n = 1,571) of cases. Diarrhoea was the most frequent complication (16.0%; n = 717 cases) followed by stomatitis (13.9%; n = 622), keratoconjunctivitis (9.3%; n = 418), hepatitis (8.7%; n = 391), pneumonia (7.8%; n = 349), respiratory insufficiency (6.3%; n = 282), otitis media (4.6%; n = 206), thrombocytopenia (3.2%; n = 143), seizures (0.2%; n = 11), and other complications (2.9%; n = 129). The highest frequency of complications (38.5%; n = 1,180) was seen in the age group over 20 years (Table 2).

**Table 2:** Number and percentage of complicated cases and hospitalisations by age group, measles outbreak, Italy, January−August 2017

Age group	Number of cases	Complicated cases (at least one complication)		Cases with more than one complication		Cases hospitalised	
		n	%	n	%	n	%
< 1 year	253	63	24.9	34	13.4	140	55.3
1–4 years	543	159	29.3	87	16.0	246	45.3
5–19 years	614	169	27.5	78	12.7	201	32.7
> 20	3,065	1,180	38.5	726	23.7	1,345	43.9
**Total**	**4,475^a^**	**1,571**	**35.1**	**925**	**20.7**	**1,932^b^**	**43.2**

^a^ Information on age was not available for two complicated cases.

^b^ Information on age was not available for one hospitalised case.

Two cases of encephalitis were reported, one in a 37-year-old adult, and the other in a one-year-old child.

Three deaths due to respiratory insufficiency occurred among children aged 16 months, 6 years and 9 years respectively. All were unvaccinated and one child was immunocompromised due to ongoing chemotherapy for a malignancy. Measles was laboratory confirmed in all three children.

Overall, 43.2% (n = 1,933) of cases were hospitalised and an additional 22.4% (n = 1,005) consulted an emergency department.

## Public health measures

In January, national authorities immediately informed all regional coordinators of the increased number of cases being reported, asking them to intensify surveillance and investigation of outbreaks. They issued two circulars; the first one was intended to remind all regional and local health authorities of the control measures recommended by the national measles elimination plan (including informing family physicians, paediatricians, gynaecologists, hospital and emergency-room physicians of the outbreak, vaccinating susceptible contacts from 6 months of age, administering immunoglobulins to susceptible contacts at high risk of complications, such as pregnant women, immunocompromised subjects and infants below 6 months of age, implementing supplementary vaccination activities, putting in place isolation protocols and infection-control measures, and distributing communication materials for HCWs and the population), while the second gave more specific recommendations regarding the administration of immunoglobulins [3,4].

Some regional health authorities released their own specific guidelines which included: (i) for general practitioners and paediatricians, the recommendation to limit referral of cases to hospitals only to those cases presenting symptoms or signs of a measles complication, in order to prevent nosocomial transmission; (ii) for hospitals, verifying the immunity status of all HCWs and vaccinating susceptible HCWs.

ISS publishes a monthly measles report and the frequency was increased to weekly bulletins to provide timely information about the measles situation in the Regions. These were published on the websites of the Ministry of Health and the ISS (EpiCentro portal), in both Italian and English languages [5,6].

The United States (US) Centers for Disease Control and Prevention (CDC) in Atlanta issued a warning for travellers to Italy on 17 April 2017 [7]. CDC recommended that travellers to Italy make sure they are protected against measles. More specifically, they recommended one dose of measles vaccine for infants aged 6–11 months and two doses for children aged 1 year or older.

## Discussion

The main reason for this outbreak is an accumulation of a large pool of measles-susceptible population due to sustained low uptake of measles vaccine in Italy over the years. Measles vaccine uptake was very low in the years following its introduction in Italy in 1976 and this has led to large vaccination gaps among adolescents and young adults, and a constantly increasing median age of reported cases [8]. The national measles elimination plan of 2011 called for supplementary immunisation activities among children over 2 years of age and susceptible groups, but these have been scarcely implemented.

Although vaccine uptake for the first dose improved after implementation of the first national elimination plan in 2003, reaching 90.6% in 2010, the target of 95% was never reached. Uptake remained stable at around 90% until 2013 but since then has decreased to 85.3% in 2015, leading to a further increase in the pool of susceptible children below four years of age, the age group with the highest incidence in the current outbreak [9]. Second–dose coverage at 6 years of age is also currently below 90% [9].

Decreased uptake of measles-mumps-rubella (MMR) vaccine in Italy in recent years is the result of vaccine hesitancy [10]. In July 2017, the Italian government approved a law [11] extending the number of mandatory vaccinations in persons up to 16 years of age. As of September 2017, proof of vaccination against 10 vaccine-preventable diseases or of having booked appointments with the local vaccination service to receive any missing vaccinations by 10 March 2018, will be required to attend kindergarten and nurseries. Older children attending elementary or middle school will also be required to present proof of vaccination; lack of compliance will not limit their access to school, but parents refusing vaccination will be required to undergo an interview by the local health authorities and to explain their reasons for not vaccinating. Following the interview, financial sanctions will be applied to families who continue to refuse to vaccinate their children.

In 2016–17, measles outbreaks have also been reported in other European countries [12]. Romania reported over 7,000 cases and 31 deaths from January 2016 to June 2017, most of them in small children [12]. On the contrary, in Italy over 70% of cases were among adolescents and young adults. The age differences in the two countries may be explained by the different levels of vaccine uptake after vaccine introduction. Currently, uptake is comparable in the two countries; however, contrary to Italy, Romania had very high vaccine uptake (> 95%) up to 2010 so many adults are protected [13].

Among the young adults affected in the current measles outbreak there are a large number of healthcare workers, and, as in previous outbreaks in Italy, nosocomial transmission was an important transmission setting [14-16]. In Italy, MMR vaccine is not required for employment as a HCW. Besides improving vaccination uptake among HCWs, there is an urgent need for implementing isolation protocols and infection control guidelines in all healthcare waiting rooms [14]. Maintaining a high index of suspicion of measles in patients with rash illness is also crucial [14,15].

The percentage of hospitalised cases in this outbreak is very high, as was the case in Wallonia, Belgium [17]; this may be due to some degree of under-reporting of milder cases who either did not seek medical care or who were not reported by primary care physicians. Also, the transmission setting was known for only one third of cases reported so the percentage of infections transmitted in hospitals may be even higher than indicated and other transmission settings (such as workplaces, public gatherings) may not have been identified. Contrary to other measles outbreaks reported in Italy in recent years and more recently in other European countries [18,19], the Roma/Sinti population was not particularly affected in this outbreak. Only 47 cases reported, 1% of the total, belonged to this group.

In addition to illness and death caused by the disease, measles outbreaks require considerable healthcare resources for evaluating cases and implementing control measures. Furthermore, the health warning by the US CDC highlights possible implications of such outbreaks beyond the immediate public health concerns in a specific country or region.

In conclusion, the size of the described outbreak highlights that there are wide measles immunity gaps in the Italian population, which together with nosocomial transmission are challenges to elimination. The new vaccination law in Italy will hopefully close immunity gaps in persons up to 16 years of age but further efforts will be necessary to reach other susceptible groups, including young adults and HCWs, through country-specific tailored strategies, and to further strengthen surveillance and outbreak response [20].
